# Iron Absorption from Three Commercially Available Supplements in Gastrointestinal Cell Lines

**DOI:** 10.3390/nu9091008

**Published:** 2017-09-13

**Authors:** Francesca Uberti, Vera Morsanuto, Sabrina Ghirlanda, Claudio Molinari

**Affiliations:** 1 Laboratory of Physiology, Department of Translational Medicine, University of Eastern Piedmont, via Solaroli 17, 28100 Novara, Italy; vera.morsanuto@med.uniupo.it (V.M.); claudio.molinari@med.uniupo.it (C.M.); 2 noiVita s.r.l.s. Spin-Off of University of Eastern Piedmont, via A. Canobio 4/6, 28100 Novara, Italy; info@noivita.it

**Keywords:** iron metabolism, iron mechanisms, Caco-2 permeability, GTL-16 permeability, DMT1 receptor

## Abstract

This study compares the absorption characteristics of two iron-based dietary supplements and their biocompatibility to bisglycinate iron, a common chelated iron form. The Caco-2 cell line—a model of human intestinal absorption—and GTL-16 cell line—a model of gastric epithelial cells—were used to perform the experiments; in the first experiments, the kinetics of absorption have been evaluated analyzing the divalent metal transporter 1 (DMT1) expression. Three different iron combinations containing 50 µM iron (named Fisioeme^®^, Sideral^®^ and bisglycinate) were used for different stimulation times (1–24 h). After this, the effects of the three iron formulations were assessed in both a short and a long time, in order to understand the extrusion mechanisms. The effects of the three different formulations were also analyzed at the end of stimulation period immediately after iron removal, and after some time in order to clarify whether the mechanisms were irreversibly activated. Findings obtained in this study demonstrate that Fisioeme^®^ was able to maintain a significant beneficial effect on cell viability compared to control, to Sideral^®^, and to iron bisglycinate. This observation indicates that Fisioeme^®^ formulation is the most suitable for gastric and intestinal epithelial cells.

## 1. Introduction

Iron deficiency anaemia is the most common nutritional disorder in the world. This condition affects a large number of children as well as women in reproductive age both in developing countries and in industrialized ones. Moreover, iron deficiency affects hemodialysis patients receiving erythropoietic stimulators as well. The numbers are impressive; 2 billion people, over 30% of the world’s population, are anaemic, over 50% due to iron deficiency, and, in poorer areas, this is frequently exacerbated by infectious diseases [1]. Several factors can contribute to iron deficiency, but low bioavailability of iron in the diet is one of the most important [2]. Iron in the diet is present both in its non-heme and heme forms, and the two of them are absorbed in duodenum. Non-heme iron accounts for more than 85% of the total iron in the diet, but it features a low bioavailability (2–7%) since several dietary factors strongly interfere with it [3]. Presently, iron supplements are the best options for maintaining iron stores in the body. However, not only the iron content, but also the bioavailability of iron for absorption largely depends on the dietary components [4]. For example, iron in its heme form is highly bioavailable, and meat-containing diets show beneficial effects as well [5]. Recently, the attention of the researchers has focused on the absorption mechanisms in the gut. Inorganic iron absorption, indeed, requires multiple mechanisms for entry and exit from duodenal and jejunal epithelial cells. If inorganic iron from the diet or supplements is not presented in a highly absorbable formulation, it will not be optimally absorbed by the intestine and subsequently transferred into the bloodstream. The part of iron trapped in the intestinal epithelial cells is then eliminated through the stools after the end of the life cycle of the enterocyte [6]. Therefore, several approaches have been attempted to improve the availability and absorption of iron through the gastrointestinal barrier. Most iron supplements are made up of ferrous salts, which is positively charged iron associated with its negatively charged counter-ions. Most common counter-ions are glycinate, sulfate, gluconate, and fumarate. Once ingested, the acidic juice within the stomach acts to dissolve the iron salt. Unfortunately, iron dietary supplementation is associated with potentially dangerous side effects and overload risk. It is well known that iron overdose can cause severe corrosive lesions to the upper gastrointestinal tract, including necrosis of the mucous membrane, ulcer and ischemia. However, epithelial gastrointestinal lesions in patients receiving iron therapy have received little attention despite its extensive use.

The aim of this study was to analyze the differences between different iron supplements in order to understand how to plan better food supplementation.

## 2. Materials and Methods

### 2.1. Cell Culture

The human intestinal Caco-2 cell line, purchased from American Type Culture Collection (ATCC, Manassas, VA, USA), was used as an experimental model [7] to predict the features of intestinal absorption following oral intake [8]. This is a widely accepted cellular model to study absorption, metabolism, and bioavailability of drugs and xenobiotics. Furthermore, this cell line has been used in other studies on iron bioavailability [9]. These cells were grown in Dulbecco’s Modified Eagle’s Medium/Nutrient F-12 Ham (DMEM-F12, Sigma-Aldrich, Milan, Italy) containing 10% fetal bovine serum (FBS, Sigma-Aldrich), 2 mM l-glutamine (Sigma-Aldrich), and 1% penicillin-streptomycin (Sigma-Aldrich) at 37 °C in incubator at 5% CO_2_. Cells were used from passages 46 to 49 to perform different experiments, such as 3-(4,5-Dimethylthiazol-2-yl)-2,5-Diphenyltetrazolium Bromide (MTT), plating 1 × 10^4^ cells in 96-well plates, Western blot and interleukin 8 (IL-8) plating 1 × 10^6^ cells in 6-well plates, plating 1 × 10^6^ cells in 6-well plates, 0.5 × 10^4^ cells were placed in Culture Slide (BD Biosciences, Bedford, MA, USA) with 4 chambers to perform iron histochemistry studies, absorption study plating 2 × 10^4^ cells on 6.5 mm transwell with 0.4 μm pore polycarbonate membrane insert (Sigma-Aldrich) in a 24-well plate. Before the experiments, cells were washed and incubated for 8 h in DMEM without red phenol and supplemented with 0.5% FBS, 2 mM l-glutamine, and 1% penicillin-streptomycin at 37 °C in an incubator and then stimulated. Cells plated on transwell insert were maintained in complete medium changed every other day, first basolaterally and then apically for 21 days before the stimulations.

GTL-16 cell line, donated by the Laboratory of Histology of the University of Eastern Piedmont, is a clonal line derived from a poorly differentiated gastric carcinoma cell line [10] widely used as a model of gastric epithelial cells. Cells were cultured in Dulbecco’s Modified Eagle Medium (DMEM) supplemented with 10% foetal bovine serum (FBS), 1% penicillin-streptomycin in incubator at 37 °C, 5% CO_2_ [11]. This cell line was plated at different densities, 1 × 10^4^ cells were plated on 96-well plates to study cell viability (MTT test); 2 × 10^4^ cells were seeded onto 6.5 mm translucent polyethylene terephthalate (PET) transwell insert 0.4 μm in a 24 well to study absorption; 0.5 × 10^4^ cells were placed in Culture Slide (BD Biosciences, Bedford, MA, USA) with 4 chambers to perform immunohistochemistry tests. Moreover, the cells were plated on 60 mm dishes until confluence to analyze the intracellular pathways through Western blot analysis and IL-8. The cells plated on transwell insert were maintained in complete medium changed every other day, first basolaterally and then apically for 7 days before the stimulations. Before stimulations, cells were synchronized by incubation in DMEM without red phenol and FBS and supplemented with 1% penicillin/streptomycin, 2 mM l-glutamine and 1 mM sodium pyruvate in an incubator at 37 °C, 5% CO_2_, and 95% humidity for 18 h [11].

### 2.2. Experimental Protocol

These cell lines were used to evaluate the absorption of iron through stomach and intestinal epithelial cells, in order to clarify the effects after oral intake. In addition, the role of oxidative stress as a consequence of treatment was investigated both during and further to the removal treatment. Finally, since the effectiveness of iron after oral intake depends on its composition, three different formulations of iron were analyzed.

The study was divided into three parts: in the first part, the kinetics of absorption, by DMT1 expression analysis, were evaluated. Both cell lines were treated with the same iron concentration (50 μM) [12] prepared in 3 different ways (named Fisioeme^®^, Sideral^®^ and bisglycinate) for different times (ranging from 1 h to 24 h). In the second part, the effects of iron prepared in the same 3 formulations in a short (3 h) and long time (24 h) were assessed, in order to understand the extrusion mechanisms. Finally, in the third part, the effects of iron, prepared in the same 3 formulations, were analyzed at the end of stimulation period and after its removal, at short (3 h plus 3 h) and long time (24 h plus 24 h) in order to clarify whether the mechanisms were irreversibly activated.

### 2.3. Agent Preparations

Fisioeme^®^ (FIS) and Sideral^®^ (SID) are dietary supplements. FIS combines the properties of iron bysglicinate, folic acid and vitamin C to improve iron absorption while avoiding such negative gastric effects such as hyperacidity. Each tablet is composed of 30 mg iron, 80 mg vitamin C, and 400 μg folic acid. On the other hand, SID is composed of iron encapsulated in a membrane. In detail, iron is associated with vitamin C and B12. Each capsule is composed of 14 mg iron, 60 mg vitamin C and 370 μg vitamin B12. FIS and SID were grinded and dissolved directly in the DMEM without red phenol and FBS but supplemented with 1% penicillin/streptomycin, 2 mM l-glutamine and 1 mM sodium pyruvate to make a 20× concentration and then diluted in the same medium to obtain 1× (50 μM iron). Finally, iron bisglycinate (BIS) formulated in pure powder was prepared directly in DMEM without red phenol and FBS but supplemented with 1% penicillin/streptomycin, 2 mM l-glutamine and 1 mM sodium pyruvate according to the solubility to obtain a concentration of 20× and then diluted in the same medium to use 1× (50 μM iron).

### 2.4. MTT Test

MTT-based In Vitro Toxicology Assay Kit (Sigma-Aldrich) was performed as described in literature [11] to determine cell viability after stimulations. Briefly, at the end of treatments, Caco-2 and GTL-16 cells were incubated with 1% MTT dye in DMEM without red phenol 0% FBS for 2 h at 37 °C in incubator [13] and cell viability was determined measuring absorbance at 570 nm with correction at 690 nm through a spectrometer (VICTORX4 multilabel plate reader, PerkinElmer, Waltham, MA, USA). Cell viability was obtained comparing the results to control cells (baseline 0).

### 2.5. Caco-2 Permeability Assay

After 21 days, the three different iron formulations were added to culture medium under different pH conditions, as reported in literature [14,15]; pH 6.5 preparations were added to the apical side, whereas pH 7.4 to the basolateral side. The slightly acidic pH (pH 6.5) in the apical side represents the average pH in the lumen of the small intestine, whereas the neutral pH (pH 7.4) in the basolateral side mimics the pH of the blood. During treatments the cells were maintained in incubator at 5% CO_2_ and, at the end of stimulations, the iron quantity was measured by a specific kit.

### 2.6. GTL-16 Permeability Assay

After 7 days, to study the effects on iron absorption of apical-to-basolateral (Ap–Bl) pH gradients, the medium was changed on both the apical (donor compartment) and basolateral (receiver compartment) sides adding HCl to the medium to obtain pH 3 at apical side for 60 min, as reported in literature [16,17]. At the end, the stimulations were performed in the same manner and conditions as previously described and then iron quantity was measured by kit.

### 2.7. Apparent Permeability Coefficient (Papp)

The presence of iron was assessed in the apical and basolateral compartments converting the amount of total volume in relationship with the surface area of the transwell (μg/cm^2^), following a classic method [18]. Briefly, the Papp (cm/s) was calculated as:

P_app_ = dQ/dt × 1/m_0_ × 1/A × V_Donor_

dQ: amount of substance transported (nmol or μg);

dt: incubation time (sec);

m0: amount of substrate applied to donor compartment (nmol or μg);

A: surface area of transwell membrane (cm^2^);

V_Donor_: volume of the donor compartment (cm^3^).

Negative controls without cells were tested to exclude transwell membranes influence.

### 2.8. Iron Quantification Assay

Iron Assay Kit (Sigma-Aldrich) measures ferrous iron (Fe^2+^), ferric iron (Fe^3+^), and total iron (total iron – ferrous iron) in samples following the manufacturer’s instructions. Briefly, Caco-2 and GTL-16 cells at apical side were lysed in 4 volumes of cold Iron Assay Buffer, centrifuged at 13,000 rpm for 10 min at 4 °C and supernatants were measured. The medium at the basolateral side was directly centrifuged at 13,000 rpm for 10 min at 4 °C and supernatants were measured. To measure total iron, 5 μL of Iron Reducer to each of the sample wells to reduce Fe^3+^ to Fe^2+^ were added. All reactions were incubated for 30 min at room temperature (RT), protected from light and then 100 μL of Iron Probe were added to each well containing standard or test samples and incubated for 60 min at RT and protected from light. The absorbance at 593 nm (A593) was measured by spectrometer (Victor). Total iron (Fe^2+^ + Fe^3+^) concentrations can be determined from the standard curve. Fe^3+^ is equal to total iron (sample plus iron reducer) −Fe^2+^ (sample plus assay buffer). The iron concentration was expressed as ng/μL.

### 2.9. Iron Histochemistry

Perls’ and Turnbull’s stains were used to visualize ferric iron (Fe^3+^) and ferrous iron (Fe^2+^), respectively [19,20] (see Appendix A).

### 2.10. IL-8 Assay Kit

Human IL-8 Quantikine ELISA Kits (R&D Systems, Abingdon, UK) [21] were used for sandwich ELISA experiments. Each condition was tested in triplicates according to the manufacturer’s specifications, and the output was measured using a microplate reader (Victor) at 450 nm within 30 min with correction to 540 nm. Concentrations (ng/mL) were obtained by fitting data to a standard curve and results expressed as a mean ± standard deviation (SD) (% vs. control).

### 2.11. Western Blot of Cell Lysates

Caco-2 and GTL-16 cells were lysed in ice Complete Tablet Buffer (Roche, Milan, Italy) supplemented with 2 mM sodium orthovanadate, 1 mM phenylmethanesulfonyl fluoride (PMSF; Sigma-Aldrich), 1:50 mix Phosphatase Inhibitor Cocktail (Sigma-Aldrich) and 1:200 mix Protease Inhibitor Cocktail (Calbiochem, San Diego, CA, USA) and 35 μg of proteins of each sample were resolved on 8% and 15% SDS-PAGE gels. Polyvinylidene difluoride membranes (PVDF, GE, Healthcare Europe GmbH, Milan, Italy) were incubated overnight at 4 °C with specific primary antibody: anti-annexin V (1:2000; Sigma-Aldrich), anti-p53 (1:250, Santa Cruz Biotechnology, Heidelberg Germany), anti-ferroportin (1:250, Santa Cruz Biotechnology), anti-ferritin (1:250, Santa Cruz Biotechnology) and anti-DMT1 (1:250, Santa Cruz Biotechnology). Protein expression was normalized and verified through β-actin detection (1:5000; Sigma-Aldrich) and expressed as mean ± SD (% vs. control).

### 2.12. Statistical Analysis

For each experimental protocol, at least four independent experiments were run; the results are expressed as means ± SD of independent experiments performed on four technical replicates. One-way ANOVA followed by Bonferroni post hoc test were used for statistical analysis, and pairwise differences compared by Mann–Whitney U tests. *p*-values < 0.05 were considered statistically significant.

## 3. Results

### 3.1. Time-Course Study on DMT1 Expression

**Figure 1:**
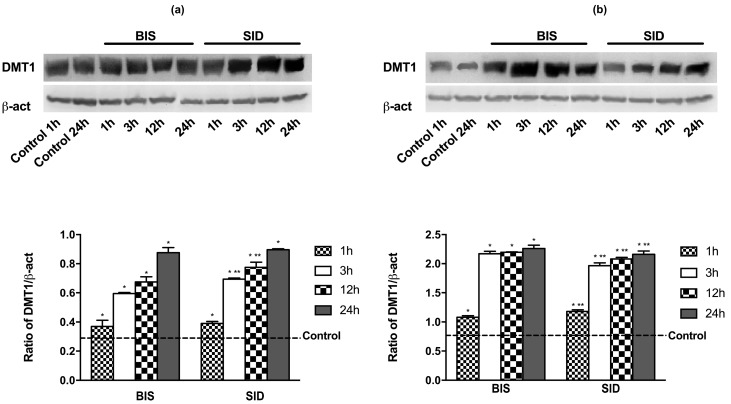
Time-course study (from 1 h to 24 h) of divalent metal transporter 1 (DMT1) receptor in GTL-16 (**a**) and Caco-2 cells (**b**) treated with different iron formulations. Western blot (upper) and densitometric analysis (down) normalized through β-act are reported. The results are expressed as means ± standard deviation (SD) of four independent experiments for each cell type. The control line shows the average of all control measures recorded at different times. BIS = iron bisglycinate; SID = Sideral^®^. * *p* < 0.05 vs. control (reported as line); ** *p* < 0.05 vs. correspondent column at different times.

The effects on DMT1 expression of 50 μM iron were analyzed by the stimulation of Caco-2 and GTL-16 cultures with SID and BIS for different times (ranging from 1 h to 24 h). Since iron formulated in FIS is a combination of BIS with other substances, in these experiments, BIS was used as a control substance. As reported in a time course study (Figure 1), the effect on DMT1 expression on both cell types showed a time dependent increase that was similar using SID to when BIS was used. The effects on DMT1 expression were significantly increased (*p* < 0.05) starting from 3 h after treatments with SID and BIS on both cell types and the maximum effects were observed at 24 h. For this reason, 3 h and 24 h were used for all successive experiments.

### 3.2. Cell Viability and Cell Regulation

**Figure 2:**
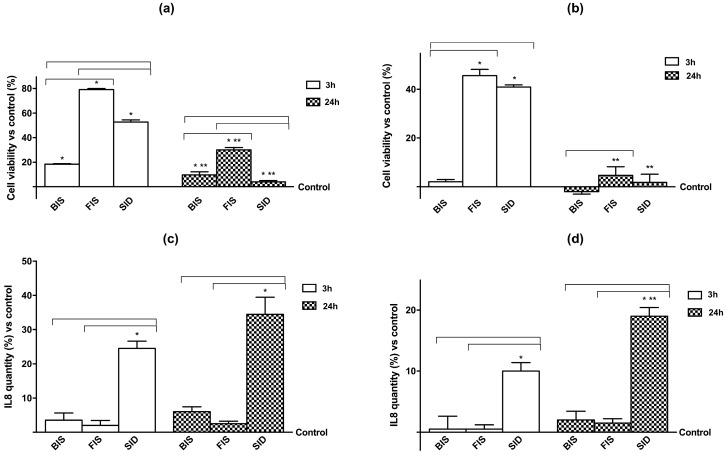
Cell viability (**a**,**b**) and interleukin 8 (IL-8) concentration (**c**,**d**) at 3 h and 24 h measured on GTL-16 and Caco-2 cells treated with different iron formulations. In A and B cell viability, measured by 3-(4,5-Dimethylthiazol-2-yl)-2,5-Diphenyltetrazolium Bromide (MTT) assay, and in C and D IL-8 concentration, measured by ELISA kit, on both cell types were reported. Data are expressed as means ± SD (% vs. control) of four independent experiments for each cell type. FIS = Fisioeme^®^. * *p* < 0.05 vs. control (reported as line); ** *p* < 0.05 vs. correspondent column at different time; bars *p* < 0.05 among different treatments at the same time.

In order to demonstrate the safety of iron supplementation, we evaluated if the levels of the investigated compounds (50 μM iron) were toxic to Caco-2 and GTL-16 cells by conducting cell viability experiments. Iron showed a formulation-dependent effect on cell viability (Figure 2A,B) on both cell types in a time-dependent manner. FIS and SID at 3 h were able to increase (*p* < 0.05) cell viability in GTL-16 cells compared to control and to BIS, confirming their beneficial properties regarding its metabolization, but, at 24 h, only FIS was able to maintain a significant beneficial effect on cell viability compared to control, to SID, and to BIS. This observation indicates that FIS formulation was more suitable for stomach epithelial cells. Moreover, in Caco-2 cells, these effects were more evident, and the differences between 3 h and 24 h obtained from each iron formulation were higher. BIS did not induce any significant change independently from stimulation time (*p* > 0.05), whereas FIS showed the maximum effect on cell viability at 3 h and maintained a minimal effect at 24 h. Finally, SID induced a significant effect at 3 h, but, at 24 h, its effect was decreased and resulted similar to the control (*p* > 0.05). These data confirm the best biocompatibility of iron formulated in FIS during transit time in the intestinal tract. In addition, as reported in Figure 2C,D, the inflammatory marker IL-8 was also investigated to verify the effectiveness of the treatments on both cell types; indeed, the concentration of IL-8 was time-dependent. In particular, in GTL-16 cells, only the sample treated with SID showed a significant increase in IL-8 concentration already at 3 h (*p* < 0.05 versus control), and this effect appeared amplified at 24 h. Similarly, SID formulation increased IL-8 concentration in Caco-2 cells as well. These data confirm the effectiveness and tolerability of FIS compared to SID on both cell types over time. SID induces the inflammatory mediator IL-8 but FIS does not, and, therefore, it may be more suitable as an iron supplement.

**Figure 3:**
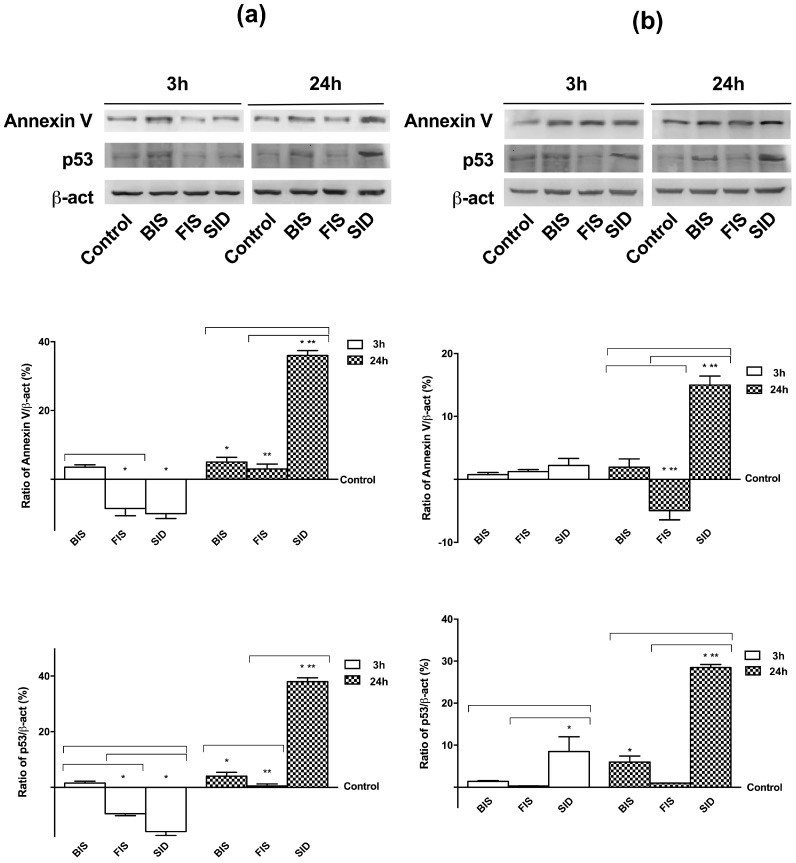
Western blot and densitometric analysis of Annexin V and p53 on GTL-16 (**a**) and Caco-2 cells (**b**) treated with different iron formulations at 3 h and 24 h. Western blot (upper) and densitometric analysis (down) are reported. The results after normalization through β-act and control, are expressed as means ± SD (% vs. control) of four independent experiments for each cell type. * *p* < 0.05 vs. control (reported as line); ** *p* < 0.05 vs. correspondent column at different time; bars *p* < 0.05 among different treatments at the same time.

In order to confirm this hypothesis, activation of p53 and Annexin V was also investigated in both cell types at 3 h and 24 h after treatment with different iron formulations (Figure 3). In GTL-16 cells at 3 h and 24 h, BIS did not induce any significant change on p53 and Annexin V activation compared to control. On the contrary, FIS and SID were able to reduce p53 and Annexin V levels compared to control (*p* < 0.05) at 3 h; however, only FIS at 24 h was able to maintain the activation at a basal level on both proteins. Indeed, at 24 h, SID significantly (*p* < 0.05) increased the activation of p53 and Annexin V. Similarly, in Caco-2 cells, both BIS and FIS were able to maintain both proteins at low level (*p* > 0.05) at 3 h and 24 h compared to control. On the contrary, SID induced activation of p53 already at 3 h (*p* < 0.05 compared to control); at 24 h, both levels of protein were augmented (*p* < 0.05 compared to control).

All of these data confirm a better influence on cell integrity exerted by the iron formulation contained in FIS compared to the other tested formulations.

### 3.3. Iron Deposition and Quantification

**Figure 4:**
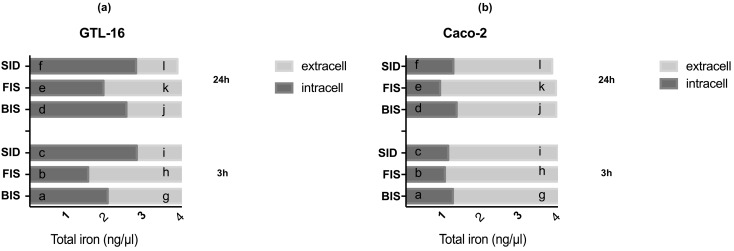
Total iron quantification on intracellular and extracellular environments of GTL-16 (**a**) and Caco-2 (**b**) cells treated with different iron formulations at 3 h and 24 h. The graphics report the iron movements quantified. The results are expressed as means ± SD of four independent experiments for each cell type. In (**a**) a *p* < 0.05 vs. b, c; b *p* < 0.05 vs. c; e *p* < 0.05 vs. d, f; g *p* < 0.05 vs. h, i; h *p* < 0.05 vs. i, k; g *p* < 0.05 vs. j; j *p* < 0.05 vs. k; k *p* < 0.05 vs. l. In (**b**) *p* < 0.05 vs. b, c; e *p* < 0.05 vs. d, f; b *p* < 0.05 vs. e; c *p* < 0.05 vs. f; h *p* < 0.05 vs. g, i; k *p* < 0.05 vs. j, l; g *p* < 0.05 vs. j; h *p* < 0.05 vs. k; i *p* < 0.05 vs. l.

**Figure A1:**
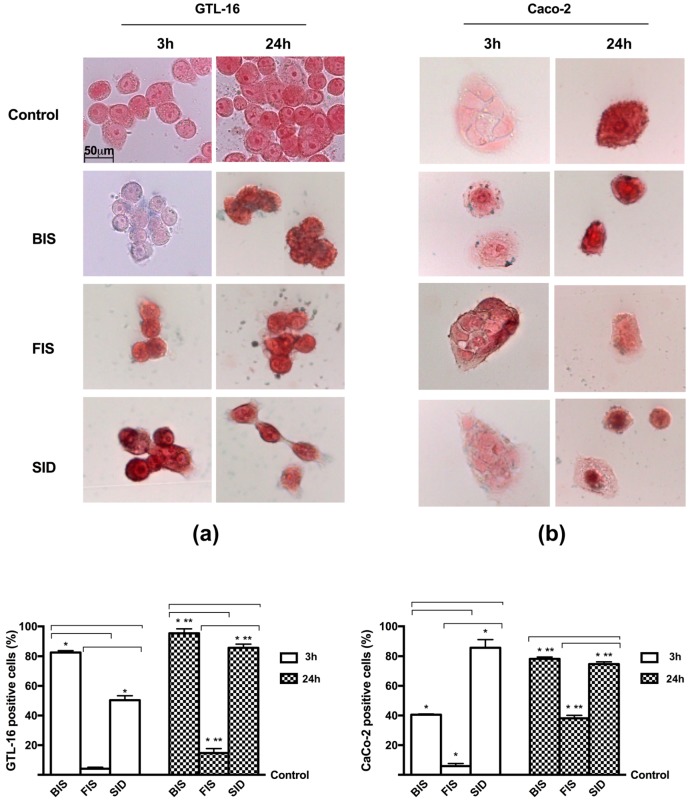
Perl’s staining on GTL-16 (**a**) and Caco-2 (**b**) cells treated with different iron formulations at 3 h and 24 h. (**top**) the images show an example of four independent experiments taken at original magnification 40×. The scale bar in the first image can be used for all others; (**bottom**) the positive cells counted were obtained from four independent experiments and expressed as means ± SD (% vs. control). * *p* < 0.05 vs. control; ** *p* < 0.05 vs. correspondent column at different times; bars *p* < 0.05 among different treatments at the same time.

**Figure A2:**
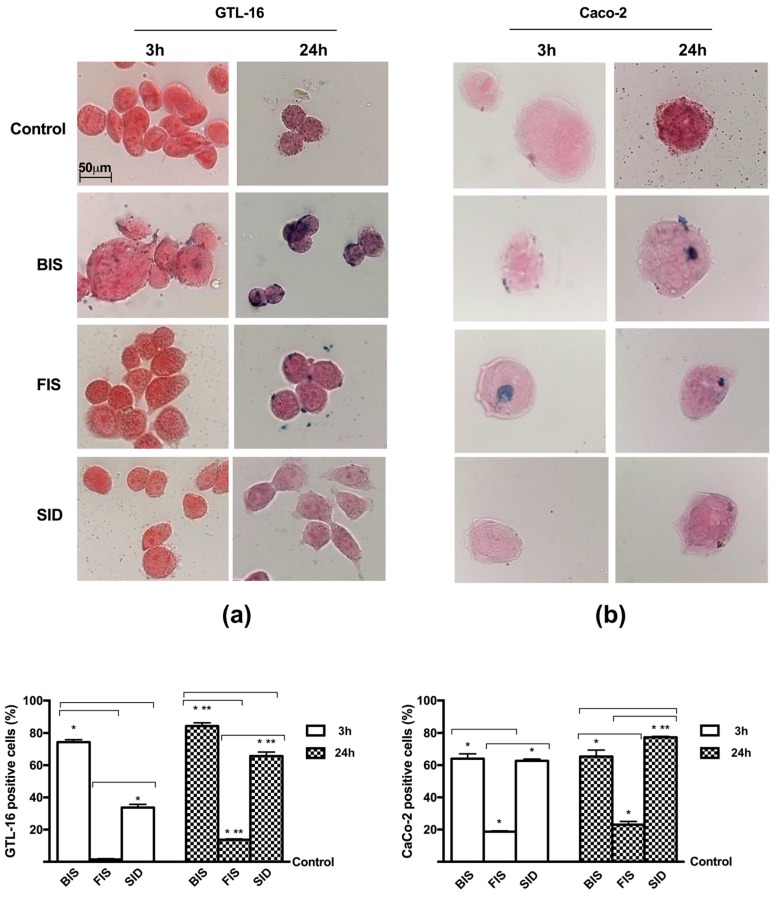
Turnbull staining on GTL-16 (**a**) and Caco-2 cells (**b**) treated with different iron formulations at 3 h and 24 h. (**top**) the images reported are an example of four independent experiments taken at original magnification 40×. The scale bar in the first image can be used for all others; (**bottom**) the positive cells counted were obtained from four independent experiments and expressed as means ± SD (% vs. control). * *p* < 0.05 vs. control; ** *p* < 0.05 vs. correspondent column at different times; bars *p* < 0.05 among different treatments at the same time.

To clarify the beneficial effects of iron supplements, some experiments were performed to evaluate the balance between Fe^2+^ and Fe^3+^ (Appendix B: Figure A1 and Figure A2) in order to verify whether Fe^3+^ was not accumulated into gastric and intestinal epithelia. Control cultures not treated with iron showed a small amount of background staining whose intensity was not different from washing solution. The slides of untreated (control) samples stained with Perls’ and Turnbull’s methods demonstrated that cells in the control condition appeared to be healthy. The intensity of staining (positive cells) for Fe^2+^ and Fe^3+^ was relatively similar regardless of time and treatments applied on both cell types; the maximum intensity was observed on both cell types at 3 h and 24 h in the presence of BIS and SID, but the differences between the two staining methods were not significant (*p* > 0.05). For this reason, on both cell types, the intracellular and extracellular iron quantity (total Fe) was also investigated (Figure 4). In GTL-16 cells, the intracellular quantity of iron (ng/μL) further to administration of BIS and FIS showed a time-dependent effect (greater at 24 h) and BIS was more present compared to FIS (*p* < 0.05). SID revealed no significant difference in time at the intracellular level. The study of the extracellular environment (iron crossing the membrane in gastric cells and in intestinal cells respectively) showed that the amount of iron was higher with FIS treatment and this effect was maintained over time. These data were confirmed by analysis of transepithelial resistance (Table 1) in which FIS exhibited a moderately higher permeability than SID and BIS both at 3 h and at 24 h. Finally, studying Caco-2 cells (Figure 4) intracellular environment, no significant changes were observed among the different iron formulations at 3 h. On the other hand, at 24 h, BIS and SID were present in greater amounts than FIS. Evaluating the extracellular environment (basolateral side, corresponding to plasma) at 3 h, no significant changes were observed. However, at 24 h, permanence time of FIS was quantitatively present longer (*p* < 0.05) compared to the other formulations. Similar data were obtained by analysis of transepithelial resistance (Table 1). Indeed, the permeability of FIS was higher than BIS and SID in both stimulation times. However, only the apical to basolateral transport was evaluated, which would not indicate whether P-gp was active against the absorption of a particular compound. In both cell types, the cell monolayers used exhibited tight junctions and this allowed the rapid permeation of the highly absorbed compound.

### 3.4. Iron Mechanisms

**Figure 5:**
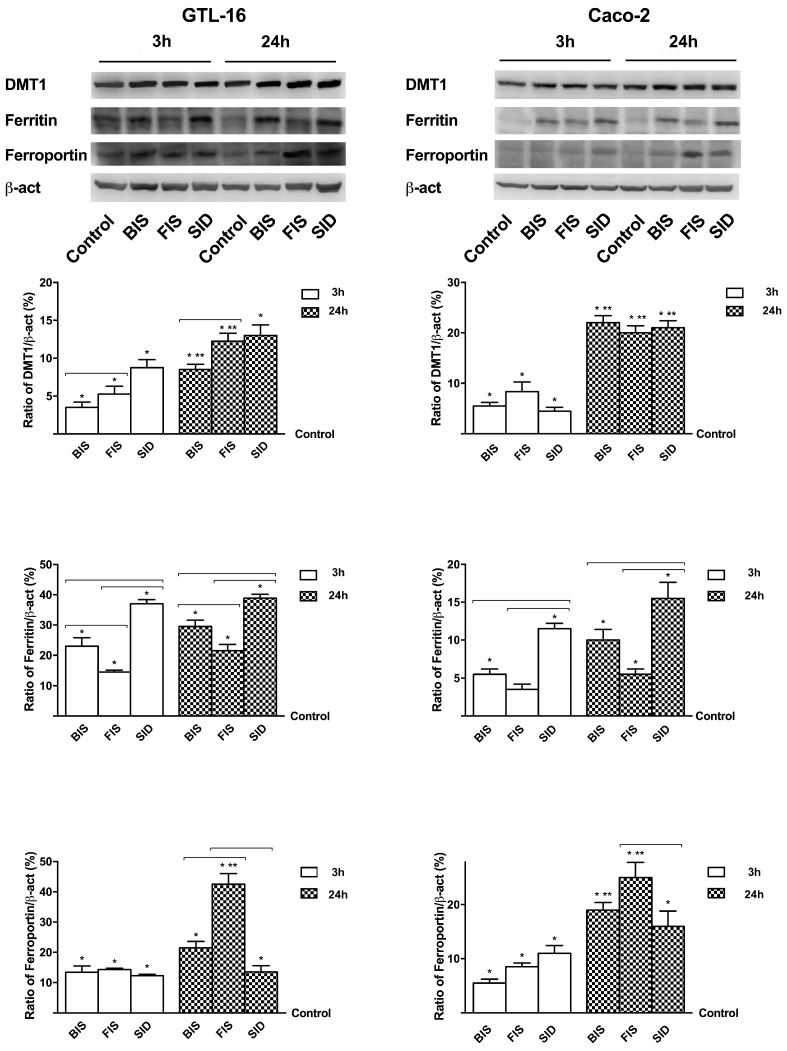
Western blot (upper) and densitometric analysis (down) of DMT1, ferritin light chain and ferroportin expressed on GTL-16 and Caco-2 cells treated with different iron formulations at 3 h and 24 h. The images are an example of four independent experiments for each cell type. The results obtained after normalization through β-act and then through control values are expressed as means ± SD of four independent experiments for each cell types. * *p* < 0.05 vs. control; ** *p* < 0.05 vs. correspondent column at different times; bars *p* < 0.05 among different treatments at the same time.

Since iron metabolism is influenced by various conditions, the importance of the different iron formulations on iron uptake (DMT1 analysis) and on transfer across the cell monolayer (ferritin light chain for transportation and ferroportin to extrude) was investigated as well (Figure 5). In GTL-16 cells, BIS was able to induce the expression of DMT1 in a time-dependent manner with a maximum effect at 24 h, but it was mainly embedded inside the cells, as shown by ferritin and ferroportin levels (*p* < 0.05 compared to control). FIS and SID were able to induce the expression of DMT1 in a similar time-dependent manner with a maximum of effectiveness at 24 h, but only FIS was able to effectively pass across the cell at 24 h (*p* < 0.05), indicating the beneficial effect of the composition in the gastric cells metabolism and into the intestinal tract. In Caco-2 cells, a similar effect on DMT1 expression among the treatments can be observed, but the formulations showed a different time-dependent extrusion mechanism with a maximum effect at 24 h. In particular, a greater transfer across the cell with FIS treatment was observed as shown by ferroportin involvement. These data demonstrate the ability of FIS to carry out its effects in a better way, compared to other iron formulations.

### 3.5. Irreversible Effects of Iron

Since the effects of iron could be negative for cells following its intracellular accumulation, some additional experiments were carried out maintaining both cell types in absence of the iron formulations for a supplementary time (3 h iron plus 3 h without iron and 24 h with iron plus 24 h without iron).

**Figure 6:**
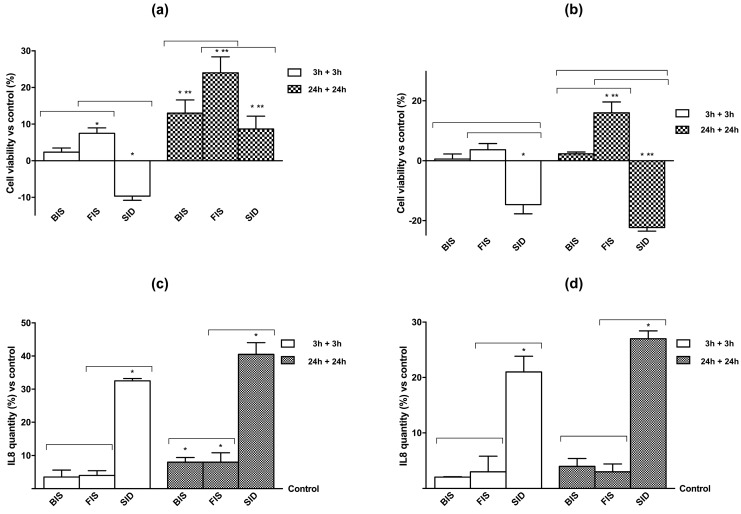
Cell viability and IL-8 concentration measured on GTL-16 and Caco-2 cells maintained for equal time (3 h plus 3 h; 24 h plus 24 h) without iron treatments. In (**a**) and (**b**) cell viability, measured by MTT assay, and in (**c**) and (**d**) IL-8 concentration, measured by ELISA kit, on both cell types are reported. Data are expressed as means ± SD (% vs. control) of four independent experiments for each cell type. * *p* < 0.05 vs. control; ** *p* < 0.05 vs. correspondent column at different time; bars *p* < 0.05 among different treatments at the same time.

As shown in Figure 6A, cell viability was time-dependent on both cell lines with a maximum effect at 24 h plus 24 h; in particular, in GTL-16 cells, BIS and FIS were able to maintain a significant cell viability (*p* < 0.05) over time, whereas SID was less effective to maintain viability. Similarly, BIS and FIS in Caco-2 cells were able to maintain viability with a maximum effect at 24 h plus 24 h (*p* < 0.05). On the other hand, SID caused a reduction of viability, thus supporting the hypothesis that iron formulated with SID could be trapped in the intestinal tract. The inflammatory framework (Figure 6B) was associated with the viability and supported the accumulation hypothesis of iron formulated with SID. Indeed, both GTL-16 and Caco-2 cells showed a significant increase in IL-8 concentration compared to control (*p* < 0.05) and to the other treatments (*p* < 0.05) already at 3 h plus 3 h.

**Figure 7:**
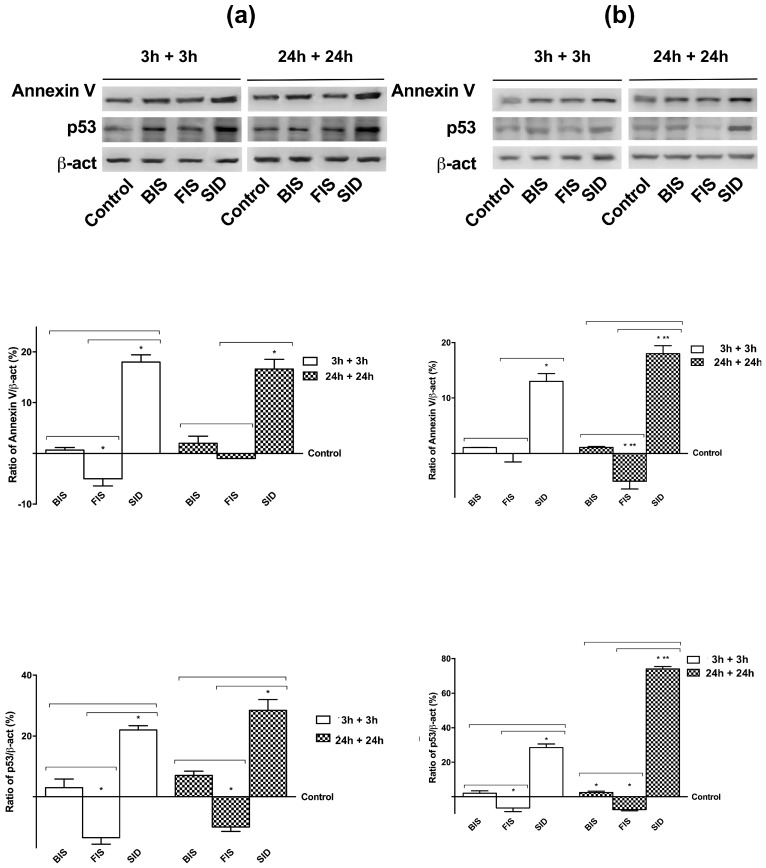
Western blot and densitometric analysis of p53 and Annexin V on GTL-16 (**a**) and Caco-2 cells (**b**) maintained for equal time (3 h plus 3 h; 24 h plus 24 h) without iron treatments. Western blot (upper) and densitometric analysis (down) are reported. The results after normalization through β-act and control, are expressed as a means ± SD (% vs. control) of four independent experiments for each cell type. * *p* < 0.05 vs. control; ** *p* < 0.05 vs. correspondent column at different time; bars *p* < 0.05 among different treatments at the same time.

In addition, the activation of p53 and annexin V (Figure 7) supported the hypothesis of the accumulation of iron associated with SID treatment on both cell types; indeed, p53 and annexin V already showed an increase at 3 h plus 3 h (*p* < 0.05).

### 3.6. Iron Metabolism

**Figure 8:**
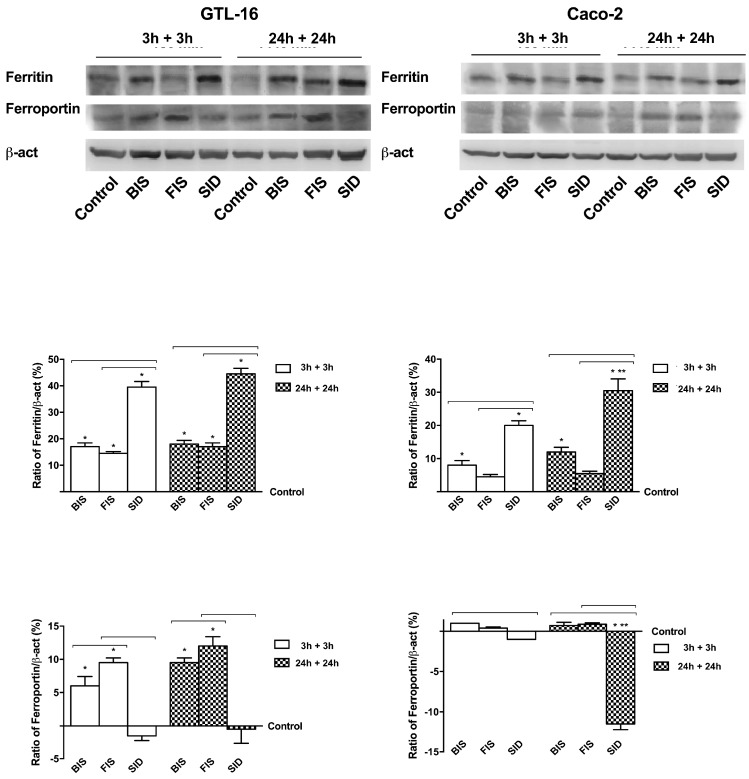
Western blot and densitometric analysis of ferritin light chain and ferroportin expressed on GTL-16 and Caco-2 cells maintained for equal time (3 h plus 3 h; 24 h plus 24 h) without iron treatments. Western blot (upper) and densitometric analysis (down) are reported. The results obtained after normalization through β-act and then through control values, are expressed as a means ± SD (% vs. control) of four independent experiments for each cell type. * *p* < 0.05 vs. control; ** *p* < 0.05 vs. correspondent column at different times; bars *p* < 0.05 among different treatments at the same time.

Since the different effectiveness of iron formulations on both cell types was demonstrated, the deposition mechanisms and the output of iron were also investigated to evaluate ferritin light chain and ferroportin in the absence of iron treatment (Figure 8).

**Figure A3:**
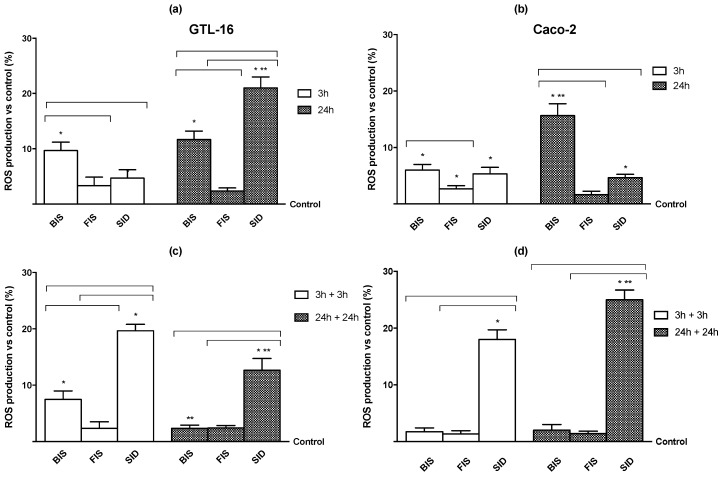
Radical oxygen species (ROS) measurements on GTL-16 (**a**,**c**) and Caco-2 (**b**,**d**) cells treated with different iron formulations at 3 h and 3 h plus 3 h or 24 h and 24 h plus 24 h. The results are expressed as means ± SD of four independent experiments for each cell type. * *p* < 0.05 vs. control; ** *p* < 0.05 vs. correspondent column at different time; bars *p* < 0.05 among different treatment at the same time.

**Figure A4:**
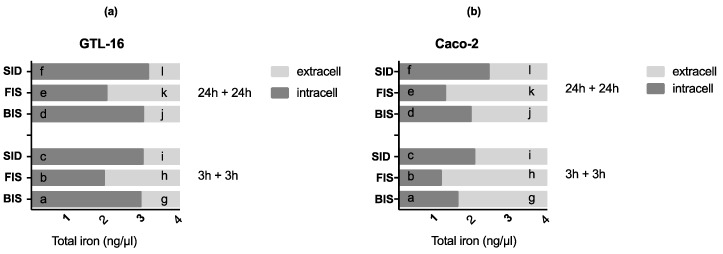
Total iron quantification in GTL-16 (**a**) and Caco-2 (**b**) cells treated with different iron formulations at 3 h plus 3 h and 24 h plus 24 h. The graphs show the iron movements quantified. Results are expressed as mean ± SD of four independent experiments for each cell type. In (**a**) a *p* < 0.05 vs. b, c, d; b *p* < 0.05 vs. c; d *p* < 0.05 vs. e; c, e *p* < 0.05 vs. f; g *p* < 0.05 vs. h, i; h *p* < 0.05 vs. i; j *p* < 0.05 vs. k; i , k *p* < 0.05 vs. l. In (**b**), a *p* < 0.05 vs. b, c, d; b *p* < 0.05 vs. c; d *p* < 0.05 vs. e, f; c , e *p* < 0.05 vs. f; g *p* < 0.05 vs. h, i, j; h *p* < 0.05 vs. i; j *p* < 0.05 vs. k, l; i *p* < 0.05 vs. l.

On both cell lines, the activation of ferritin light chain was significantly reduced in the samples treated with FIS and significantly increased (*p* < 0.05) in the samples treated with SID in a time-dependent manner with maximum effects at 24 h plus 24 h. On the contrary, the output receptor ferroportin, in GTL-16 cells was increased in a time-dependent manner in samples treated with BIS and FIS with maximum effectiveness at 24 h plus 24 h, whereas SID treatment showed a significant reduction (*p* < 0.05) on ferroportin expression. Similar mechanisms have been observed in Caco-2 cells as well, with a greater inhibition of ferroportin at 24 h plus 24 h compared to control (*p* < 0.05). These data demonstrate that iron in all formulations was partially accumulated and slowly released over time; however, this effect did not appear after SID treatment because iron was trapped within the cells. In additon, further experiments were carried out to evaluate the production of reactive oxygen species (ROS) induced both in GTL-16 and Caco-2 cells by the administration of BIS, FIS and SID (Appendix B: Figure A3). The results are comparable to those observed in viability and inflammatory framework experiments. FIS showed the lowest ROS increase in both cell cultures compared to BIS and SID, and this increase in ROS production is always not significant compared to control regardless of exposure time. Finally, in total iron quantification experiments, FIS has always shown to induce a better balance between intracellular and extracellular compartment compared to BIS and SID (Appendix B: Figure A4); indeed, the extracellular amount, which is the one that has passed through the barrier, is higher or equals the intracellular amount. Thus, FIS is better than BIS and SID in regards to this effect. This result was also observed in long exposure time and even without stimulus. Moreover, iron stores are lower in Caco-2 than in GTL-16.

## 4. Discussion

Current strategies about iron deficiency treatment are based on oral ferrous iron supplements; however, these may be poorly tolerated by patients due to gastrointestinal side effects [12,22], which interfere with the efficiency of the treatment itself. The biocompatibility of iron dietary supplements is a common issue in treatment of iron deficiency anaemia. Moreover, the iron formulation can be very important in determining such side effects as severe corrosive injury to the epithelial gastrointestinal cells, including mucosal necrosis, ulceration, and ischemia.

In this study, we compared the biocompatibility and biological activity of a range of commercially available iron supplements using a well-characterized in vitro model of digestive system [23]. Cell lines used in the present work are widely accepted as an experimental model of gastrointestinal system.

The Caco-2 human intestinal cell model is among the most widely used for drug development, toxicology and intestinal physiology studies. Caco-2 cells differ in a monolayer of polarized cells coupled by junctions that express many morpho-functional characteristics of the absorbent epithelium of the small intestine. For this reason, Caco-2 cells have been extensively used for iron uptake studies [24]. On the other hand, the GTL-16 cell line is a clonal line derived from a poorly differentiated gastric carcinoma cell line [10] and is widely used as a model of gastric epithelial cells. Findings obtained in this study demonstrate that FIS was able to maintain a significant beneficial effect on cell viability and integrity compared to control, to SID, and to BIS. Furthermore, the inflammatory framework was maintained at a more basal level by treatments with FIS and BIS than that observed after SID treatment. Similar data were observed after removal of iron. These observations indicate that FIS formulation is more suitable for gastric and intestinal epithelial cells.

It is noteworthy that tests on intracellular iron, total iron quantification, and molecular mechanisms demonstrate that FIS is able to physiologically regulate iron metabolism, whereas iron in SID appears to be trapped within cells. The epithelium of the intestinal mucosa is one of the major barriers in terms of extension between the intestine and the internal organs. As such, it is a dual target of any toxic insult from drugs or substances in the diet: in fact, mucosal alterations are not only a damage to the tissue itself but can cause uncontrolled passage of potentially toxic substances from the intestinal lumen to blood. The physiological role of FIS is further demonstrated by the small and not significant increase in ROS production. Details are reported in Appendix B. In addition, the permeability resistance observed in both cell types showed a mutual relationship between measured iron concentration and cellular permeability, which accounts for the better absorption and solubility of iron bysglicinate compared to iron pyrophosphate. In the future, additional experiments will be performed in vivo to explore the effects of these iron formulations in an anaemic mouse experimental model.

## 5. Conclusions

In conclusion, FIS that contains iron bisglycinate has a better bioavailability than compound BIS and SID, which we have investigated. For this reason, FIS is a good choice of iron preparation as a food supplement.

## Figures and Tables

**Table 1:** Apical to basolateral permeability. Apparent permeability values (Papp) represent mean data ± standard deviation (SD) from three separate measurements of each compound tested in one experiment, each with four independently analyzed.

Iron Formulation	P_app_ (10^−6^ cm/s) at 3 h		P_app_ (10^−6^ cm/s) at 24 h	
	GTL-16	Caco-2	GTL-16	Caco-2
SID	5.02 ± 1.1 **	15.07 ± 1.9 **	0.63 ± 0.035	1.57 ± 0.41
FIS	12.56 ± 1.43 *^,^**	16.08 ± 2 **	1.38 ± 0.4	1.88 ± 0.43
BIS	10.35 ± 1.82 *^,^**	13.56 ± 1.8 **	0.94 ± 0.6	1.44 ± 0.35

SID = Sideral^®^; FIS = Fisioeme^®^; BIS = iron bisglycinate. * *p* < 0.05 vs. SID; ** *p* < 0.05 between the same treatment at different time.

## Appendix A

### Appendix A.1. Method: Iron Histochemistry

The cells cultured on chamber slides at the end of stimulations were washed three times with cold PBS 1× supplemented with 2 mM sodium orthovanadate, and fixed using a cold fixative solution (3.7% formaldehyde, 3% sucrose in PBS 1×) for 20 min at RT. Then, the chamber slides were incubated in a sufficient amount of Prussian Blue cell staining reagent composed of an equal amount of potassium ferrocyanide and HCl or of Turnbull staining reagent composed of 5:1 potassium ferrocyanide and HCl for 10 min at RT. Then, the slides were counterstained with Safranin-O for 5 min at RT and mounted with Bio Mount (Bio-Optika, Milan, Italy). The number of positive cells was calculated as described elsewhere [25]. Briefly, 12 different areas (1 mm^2^) randomly selected from each section were taken, and the number of signals was determined using ImagePro 3 software (NIH, Bethesda, MD, USA). The results were expressed as means ± SD (% vs. control).

### Appendix A.2. Method: Radical Oxygen Species (ROS) Analysis

ROS production was determined as a superoxide dismutase-inhibitable reduction of cytochrome C, following a standard technique [13,20]. In all samples (both treated and control), 100 μL of cytochrome C (Sigma-Aldrich) and in another one 100 μL of superoxide dismutase (Sigma-Aldrich) were added for 30 min in an incubator. The absorbance was measured at 550 nm by spectrometer (VICTORX3 Multilabel Plate Reader, Perkin Elmer, Waltham, MA, USA) and the O_2_ was expressed as nanomoles per reduced cytochrome C per microgram of protein and reported as a mean ± SD (%).
